# Characterization and diversity of native *Azotobacter* spp. isolated from semi-arid agroecosystems of Eastern Kenya

**DOI:** 10.1098/rsbl.2021.0612

**Published:** 2022-03-23

**Authors:** Priscillah Wanjira Wakarera, Patroba Ojola, Ezekiel Mugendi Njeru

**Affiliations:** Department of Biochemistry, Microbiology and Biotechnology, Kenyatta University, PO Box 43844-00100, Nairobi, Kenya

**Keywords:** biodiversity, bioinoculant, *Azotobacter*, plant growth-promoting rhizobacteria, smallholder farmers

## Abstract

Declining food production in African agroecosystems is attributable to changes in weather patterns, soil infertility and limited farming inputs. The exploitation of plant growth-promoting soil microbes could remedy these problems. Such microbes include *Azotobacter*; free-living, nitrogen-fixing bacteria, which confer stress tolerance, avail phytohormones and aid in soil bioremediation. Here, we aimed to isolate, characterize and determine the biodiversity of native *Azotobacter* isolates from soils in semi-arid Eastern Kenya. Isolation was conducted on nitrogen-free Ashby's agar and the morphological, biochemical and molecular attributes evaluated. The isolates were sequenced using DNA amplicons of 27F and 1492R primers of the 16S rRNA gene loci. The Basic Local Alignment Search Tool (BLASTn) analysis of their sequences revealed the presence of three main *Azotobacter* species viz., *Azotobacter vinelandii, Azotobacter salinestris* and *Azotobacter tropicalis*. Kitui County recorded the highest number of recovered *Azotobacter* isolates (45.4%) and lowest diversity index (0.8761). Tharaka Nithi County showed the lowest occurrence (26.36%) with a diversity index of (1.057). The diversity was influenced by the soil pH, texture and total organic content. This study reports for the first time a wide diversity of *Azotobacter* species from a semi-arid agroecosystem in Kenya with potential for utilization as low-cost, free-living nitrogen-fixing bioinoculant.

## 1. Introduction

Modern science has focused on the positive interactions in the rhizosphere that promote plant growth and development [1]. Harnessing beneficial microbes for use as soil inoculants has proven effective in promoting plant growth. These microbes enhance germination rates; foster root development and growth; and increase crop yield, leaf area and chlorophyll content, and essential nutrient content while promoting drought tolerance [2]. The most studied aspect of plant growth-promoting rhizobacteria (PGPRs) is their ability to fix atmospheric nitrogen.

*Azotobacter* are regarded as the most dominant source of nitrogen in soils lacking symbiotic nitrogen fixers [3]. There are several species: *Azotobacter* (*A). chroococcum, A. tropicalis, A. vinelandii, A. paspali, A. nigricans, A. beijerincki, A. armeniacus* and *A. salinestris* [4], with each displaying varying chemical and biological interactions [5]. In addition to nitrogen-fixing ability, *Azotobacter* produce plant growth-promoting substances, such as phytohormones, enhance plant nutritional uptake and aid in seed germination and formation of shoots and root elongation systems [6]. They also increase the dry matter accumulation by increasing shoot and root length during growth [7]. The presence and efficiency of *Azotobacte*r in soil are influenced by various biotic and abiotic environmental factors [8], including soil physico-chemical properties. *A*z*otobacter* spp. are known to produce capsular slime in response to environmental stress. This reduces oxygen uptake into the cell, enhancing nitrogenase activity [9].

Studies on PGPRs are highly significant, especially in Africa, where farming is dominated by resource-limited smallholder farmers who cannot afford costly agrochemicals. Soil inoculation with actively living beneficial microorganisms is eco-friendly and sustainable, providing an organic and cost-efficient alternative to the use of chemical fertilizers [4]. To ensure a high-performing and efficient biofertilizer formulation, native strains are recommended due to their adaptation to the specific agroecological zone and their competitiveness over non-indigenous strains in soil [10].

Therefore, we sought to characterize native *Azotobacter* strains present in varying smallholder agricultural soils within the Eastern Kenya semi-arid ecological zone and to determine their genetic diversity. We also sought to determine the influence of soil quality on the occurrence and diversity of various *Azotobacter* strains.

## 2. Material and methods

### (a) Study site, sampling and physico-chemical analysis

Soil samples were collected from smallholder farms in semi-arid zones of Tharaka Nithi, Embu and Kitui Counties of Eastern Kenya. Soil sampling was conducted during the dry, post-harvest season in August 2019. The soil samples were obtained from 20 farms within each of the three Counties, aseptically. Each sample was collected from 20 random sampling points, obtained in a zigzag manner per farm and thoroughly mixed, creating a composite soil sample that was collected into sterile labelled khaki bags. They were air dried and sieved using 2 mm aperture sieves and stored for later use.

The soils' physico-chemical analysis was conducted by determining the soil pH and soil texture using a glass electrode pH meter and the hydrometer method, respectively. Subsequently, the soil texture was grouped as sandy-loam (SL), loamy-sandy or sandy-clay-loam (SCL). The total organic matter, total soil nitrogen and the available phosphorus contents were determined by the Walkley and Black oxidation method, the macro-Kjeldahl method and Olsen extraction method, respectively [11].

### (b) Isolation of *Azotobacter*

One gram of soil was placed in a sterile test tube and suspended in 9 ml of distilled sterile water then thoroughly agitated. Serial dilutions were prepared up to 10^−3^ and an aliquot of 10 µl of each dilution spread on a plate containing Ashby's Nitrogen-free selective media [12]. The inoculated plates were then incubated at 28°C for 5 days, followed by sub-culturing on Ashby's media to obtain pure cultures.

### (c) Characterization of isolates

#### (i) Morphological and biochemical characterization

Characterization was conducted using Gram staining and a colony feature test. This included colony colour, texture, size, shape and margins, elevation on the agar and colony form [13]. The production of pigment was evaluated by sub-culturing the isolates on modified Ashby's benzoate (0.5% w/v) medium and the subsequent pigment production was recorded after incubation at 28°C for 5 days according to Banerjee *et al*.'s [14] procedure. Acid production in the media was evaluated by growing the isolates in Ashby's media containing bromothymol blue as an indicator.

### (d) Molecular characterization

#### (i) Genomic DNA extraction

One representative isolate was selected randomly in every morphological group for DNA extraction and an additional isolate was selected from groups with a high number of individuals. Selected representative isolates were named P1 to P25. Bacterial DNA from pure cultures was extracted by use of the Zymo Quick-gDNA^TM^ Mini Prep DNA extraction kit using the manufacturer's protocol. Gel electrophoresis was conducted using 1.0% agarose gel and DNA bands were visualized on UV trans-illuminator.

### (e) Polymerase chain reaction amplification and sequencing of 16S rRNA gene

Polymerase chain reaction (PCR) analysis was conducted using universal primers 27F (5′-AGAGTTTGATCCTGGCTCAG-3′) and 1492R (5′-TACGGCTACCTTGTTACGACTT-3′), which are complimentary to the conserved regions of the bacterial 16S rRNA gene. The PCR reaction was carried out in a Techgene thermocycler (FTGENE5D model) having thermal cycling conditions set as follows: the initial denaturation step was at 95°C for 3 min, 35 cycles of denaturation at 94°C for 45 s, annealing at 51.8°C for 45 s, elongation at 72°C for 2 min and final extension step at 72°C for 5 min. The samples were finally stored at −4°C.

Gel electrophoresis of 3 µl of the PCR product mixed with 2 µl of loading dye containing SYBR green stain was loaded on 1.4% (w/v) agarose gel and electrophoresis was done at 80 V for 30 min and subsequent visualization was done on a UV trans-illuminator. The PCR products were sent to Macrogen-Netherlands for purification and Sanger sequencing using 27F and 1492R primers.

### (f) Data analysis

After base-calling and creation of a consensus sequence, the DNA sequences were compared to available sequences on the NCBI GenBank database using Basic Local Alignment Search Tool (BLASTn) software. The sequences with the highest hits on the 16S rRNA gene were extracted and aligned on the ClustalW program [15]. Further, evolutionary analyses were conducted in MEGA X software; the phylogenetic tree's evolutionary distance depiction was computed using the Jukes–Cantor method showing the number of base substitutions per site. The analysis involved 33 nucleotide sequences; codon positions included were 1st + 2nd + 3rd + noncoding. All ambiguous positions were removed for each sequence pair. There were a total of 1635 positions in the final dataset [16].

The diversity indices and evenness were calculated on PAST v.4.0 software based on the numbers of the recovered *Azotobacter* spp. in the different morphological groups. Diversity indices including Simpson_1-D and Shannon_H, the dominance and evenness were also calculated.

The influence of soil quality on the occurrence and biodiversity of the recovered *Azotobacter* isolates was determined by evaluating correlation through a redundancy analysis (RDA). This was achieved by comparing the recovered sequences' diversity index against the soil physico-chemical properties using Canoco 5 software [17].

## 3. Results

### (a) Morphological and biochemical identification of isolates

A total of 221 cultured isolates were recovered from all soil samples in the three regions, 101 of the recovered isolates were from Kitui County, 64 from Tharaka Nithi County and 56 from Embu County. The isolates' morphological and biochemical characteristics are as shown in electronic supplementary material, table S1. They were grouped into 14 groups based on their observable characteristics.

After growing on Ashby's benzoate media, all of the isolates produced a black and brown pigment except for those in morphogroups IsB, IsC, IsG, IsH, IsJ, IsK, IsL and IsM, which remained clear or cream (electronic supplementary material, figure S1 and table S2). Growth on BTB amended Ashby's media showed acid production by all morphogroups except for IsG, IsL, IsM and IsO, whose media remained green (electronic supplementary material table S1 and figure S1) by day 5 of incubation.

### (b) Molecular characteristics of the isolates

The genomic DNA was extracted from all of the 25 morphological group representative isolates and PCR of the 16S rRNA gene produced a single band of 1.5 kb. After PCR product purification and Sanger sequencing, sequence editing and alignment of all the representative isolates were carried out and compared to the known identities on the NCBI database. Phylogenetic analysis was done on MEGA X software [18]. The recovered *Azotobacter* isolates included *A. vinelandii,* which was the most common, followed by *A. tropicalis,* and the least common being *A. salinestris*. The recovered sequences were deposited in the NCBI database and their accession numbers retrieved (table 1).

**Table 1.** Phylogenetic match and relationship of isolates' sequence identity of the partial 16Sr RNA gene sequences.

isolate laboratory designation	species/strain identification	GenBank accession number	16Sr RNA gene similarity (%)	sizes of sequences	soil sample identity^a^
P1 (IsA)	*Azotobacter vinelandii* strain M-A (EJ032011.1)	MW586972	98.65	1437	Eb
P6 (IsF)	*Azotobacter vinelandii* strain AV1 (MK847515.1)	MW897943	95.2	1442	Tf
P9 (IsI)	*Azotobacter tropicalis* strain KBS (AB236160.1)	MW586876	92.6	1480	Th10
P17 (IsA)	*Azotobacter vinelandii* strain ISDS (EF620452)	MW586882	98.24	1491	Ed
P19 (IsJ)	*Azotobacter vinelandii* strain AV1 (MK847515.1)	MW586883	97.22	1405	Tf
P22 (IsE)	*Azotobacter tropicalis* strain KBS (AB236160.1)	MW586884	99.36	1480	K1
P23 (IsK)	*Azotobacter vinelandii* strain ISDS-428 (EF620447.1)	MW897942	94.55	1488	K1
P24 (IsI)	*Azotobacter tropicalis* strain KBS (AB236160.1)	MW586885	99.57	1480	Kc
P25 (IsO)	*Azotobacter vinelandii* strain (OK083775.1)	MZ066656	93.2	1488	Ec
P3 (IsC)	*Klebsiella variicola* strain VITGAJ4(MT829337.1)	MW586873	98.03	1477	T3
P5 (IsC)	*Klebsiella pneumoniae* strain 50595 (MW586874.1)	MW586874	99.29	1412	KT5
P7 (IsG)	*Mucilaginibacter limnophilus* strain YBJ-36 (NR_165720.1)	MW586875	94.47	1401	Ec
P10 (IsB)	*Paenibacillus mucilaginosus* strain 5S5 (MH179089.1)	MW586877	99.36	1411	E15
P12 (IsL)	*Massilia* sp. strain SC0-D23 (FN386766.1)	MW586878	98.06	1444	KT12
P13 (IsM)	*Ensifer* sp. strain YRG17 (MG859512.1)	MW586879	99.26	1352	Kb
P14 (IsO)	*Rhizobium* sp*.* strain SWFU-1227 (JN896883.1)	MW586880	94.57	1383	Th10
P15 (IsH)	*Mesorhizobium* sp. strain ORS3670 (JN085517.1)	MW586881	99.41	1387	Ed
P2 (IsB)	*Paenibacillus graminis* strain (JQ436907.1)	MW897940	85.84	1093	Th16
P16 (IsH)	*Stenotrophomonas rhizophila* strain R2A2_6_7 (LR722866.1)	MW897941	98.43	1415	Tc

^a^Soil samples Eb, Ed, Ec and E15, soil samples K1, Kc, KT5, KT12 and Kb soil samples Th10, Tf, T3, Th16 and Tc, were collected from Embu, Kitui and Tharaka Nithi Counties, respectively.

### (c) Phylogenetic analysis of the bacteria

**Figure 1.**
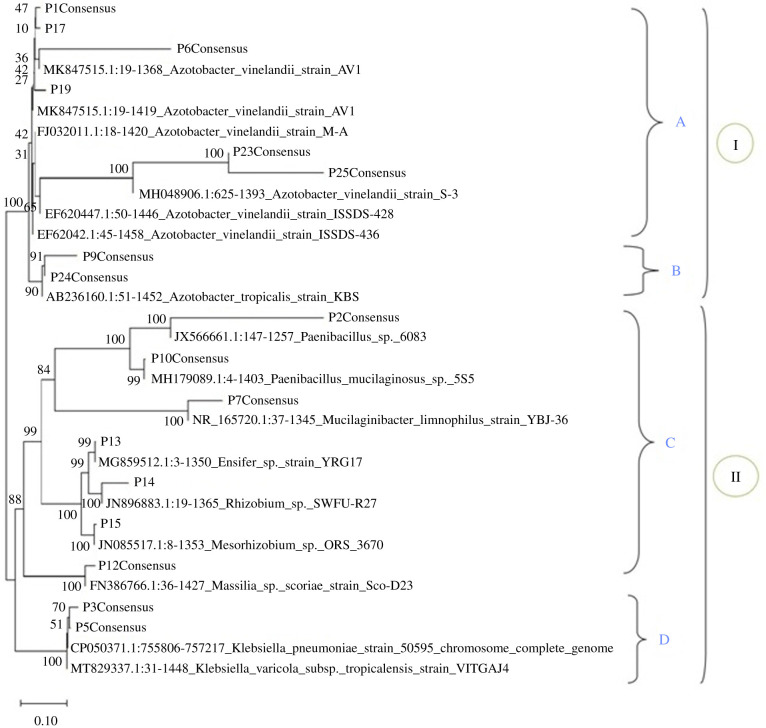
Phylogenetic tree displaying isolates' identity based on 16SrRNA gene sequence.

The evolutionary relationship of the recovered isolates was displayed in a phylogenetic tree based on the 16S rRNA gene sequences, using the Jukes–Cantor method. The isolates' sequences clustered into two main clusters (I and II) (see figure 1). Cluster I had two sub-clusters (A and B), with *A. vinelandii* isolates (P1, P17, P6, P19, P23 and P25) clustering together in sub-cluster A, while sub-cluster B contained *A. tropicalis* isolates (P9 and P24). Cluster II similarly had two sub-clusters C and D; sub-cluster C comprised alphaproteobacteria and firmicutes that include *Massilia* spp.*, Mesorhizobium* spp.*, Rhizobium* spp.*, Ensifer* spp.*, Paenibacillus* spp*, P. mucilaginosus* and *M. limnophilus.* Sub-cluster D comprised *K. variicola* and *K. pneumoniae,* which are both free-living nitrogen fixers and are gammaproteobacteria (figure 1).

### (d) Influence of soil physico-chemical properties on biodiversity

The Kitui and Embu County soils were SCL, while Tharaka Nithi County soil was predominantly SL. The lowest pH value of 5.11 was recorded in Embu County soil sample EM10, and the highest pH of 7.4 recorded from Tharaka County sample T5 (electronic supplementary material, table S3). The soils in Embu County showed the highest diversity and evenness of *Azotobacter* isolates (electronic supplementary material, figure S3). The lowest evenness (0.7193) was observed in Tharaka soils and the highest dominance (0.4936) observed in Kitui soils, which had the highest recovery percentage of individuals and least taxa (table 2; electronic supplementary material, figure S3).

**Table 2.** Diversity of *Azotobacter* spp. in Embu, Kitui and Tharaka Nithi Counties of Kenya.

	THARAKA	EMBU	KITUI
Taxa_S	4	5	3
Individuals	29	31	50
Dominance_D	0.4388	0.232	0.4936
Simpson_1-D	0.5612	0.768	0.5064
Shannon_H	1.057	1.538	0.8761
Evenness_e^H/S	0.7193	0.9313	0.8005

**Figure 2.**
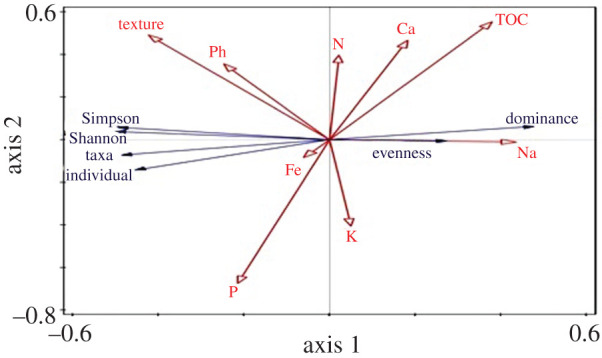
RDA showing the effect of soil characteristics on the diversity of nitrogen fixers in the soil.

Based on the RDA analysis, the soils' phosphorus and iron contents positively related to the taxa and individuals, but these were not related to the soil texture and pH (figure 2). According to the figure 2 RDA plot, dominance positively associated with TOC, Ca and N while evenness positively associated with the K and Na contents. Nitrogen had a negative correlation with Simpson, Shannon, Taxa and number of individuals of *Azotobacter* in the soil as well as evenness (figure 2).

## 4. Discussion

In this work, we sought to characterize and determine the diversity of *Azotobacter* spp. from smallholder agroecosystems in semi-arid zones of Eastern Kenya. The goal was to identify suitable strains that can be exploited as low-cost free-living nitrogen-fixing microbial inoculants. Remarkably, we recovered 221 isolates, which on further genetic analysis revealed the presence of 110 *Azotobacter* isolates.

Drawbacks in morphological *Azotobacter* identification make metabarcoding a necessity in characterization [4]. Universal primers (27F and 1492R) have been used for amplification of 16S rRNA gene for genetic identification of bacteria including *Azotobacter* [4,19]. Their pleiomorphic nature was observed during characterization; while isolates P6 and P25 (groups IsF and IsO, respectively) were translucent, glistening, gummy and produced a browning pigment (electronic supplementary material, table S1) characteristic of the *A. vinelandii* [20], isolates P1 and P17 (group IsA) produced a green pigment that darkened with age, was mucoid and could have been morphologically mistaken for *A. paspali* [21]*.* Additionally, isolates in morphogroups IsE, IsO, IsF, IsI and IsD produced brown pigmentation in Ashby's media, which is a characteristic of *A. chroococcum, A. salinestris and A. tropicalis* [13,14].

The production of brown or black pigment in the benzoate-enriched Ashby's agar (electronic supplementary material, figure S1) was used for further characterization in this study, as recommended by Martyniuk & Martyniuk [22]. The distinct green pigment-producing isolates (P1 and P17) were obtained from Embu County farms E13, Ea, Eb and Ee. The gene sequencing using amplified 16S rRNA gene loci identified them as *A. vinelandii* (EF620452.1**)** with 99.15% similarity index. This strain is found in soils containing low iron (Fe) content [20]. The four soils it was isolated from had a mean iron content of 14 ppm, which is considerably low. Fekete *et al.* [23] investigated the production of siderophore in iron-limited cultures and found that a yellow-green fluorescent peptide caused this pigmentation in culture. *A. salinestri* is found in soils containing high Fe, Na and salinity [24]. Isolates P22 and P9 identified as *A. tropicalis* had the second highest occurrence. This species is commonly found in agricultural soils in tropical areas and was distributed in soils across all the sampled Counties [25].

Interestingly, we found that soil characteristics influenced biodiversity of the microbial communities in the soil [26,27]. The sampled soils had an average pH 6.1, contained considerable amounts of organic matter and varying levels of phosphorus (P) that are beneficial for the growth of these free-living nitrogen-fixing bacteria (FNFB), as reported by Raimi *et al*. [28]. Farms in Embu County showed the lowest recovery of FNFB, possibly due to its low pH (approx. 5.68), with the highest recovery being in samples from farms in Tharaka Nithi County. Notably, studies report *Azotobacter* spp. abundance in soils with a pH above 6.5 [29], explaining the high number of isolates recovered from soils in farms Kd, Kb and Tf, which had pHs of 6.7, 6.58 and 7.1, respectively.

There were significant differences in *Azotobacter* biodiversity between farms. These can be attributed to variations in the soil characteristic, as described by Mhete *et al*. [26]. Based on RDA analysis soil texture, pH and phosphorus positively correlated with Shannon and Simpson diversity indices of the *Azotobacter* isolates, as reported in previous studies [30–32]. However, total organic carbon in the soil correlated negatively with Shannon diversity of the isolates but positively with isolate dominance, as has been documented by Chen *et al*. [33]. Soils with a high organic carbon content promote the growth of microorganisms in the rhizosphere. *Azotobacter* are found in soils with high nitrogen content due to their nitrogen fixation ability [4]. The amount of phosphorus in the soil greatly correlated with the taxa and number of the microbes. This is attributable to P solubilization by *Azotobacter* and other P-solubilizing bacteria [34]. The Na content of the soil influenced diversity the least, but showed a strong influence on their evenness [30]. The majority of the isolates were found in soils classified as SCL, attributable to their high requirement for P. These results concur with findings by Ridvan [31] on *Azotobacter* biodiversity. The utilization of native strains in bioinoculant formulation ensures adaptability to the soil. Some species have a higher affinity for specific soil characteristics and would therefore grow and produce plant growth-promoting metabolites in these conditions [30]. Therefore, the exploitation of diverse species ultimately leads to higher efficacy of the microbial inoculant contributing significantly to crop productivity.

## 5. Conclusion

This study demonstrated the presence of various native *Azotobacter* spp*.* in the semi-arid areas of Eastern Kenya, whose biodiversity was greatly influenced by the soils' characteristics. Notably, these areas are inhabited by resource-limited smallholder farmers, who could greatly benefit from this untapped biodiversity if well exploited. Moreover, the revelation of these native isolates demonstrates potential for sustainable food production through their exploitation as low-cost bioinoculants. Nevertheless, limited research and literature on *Azotobacte*r spp. occurrence in Kenyan soils are a hindrance to local utilization. Further studies should elucidate the plant growth-promotion efficiency of native *Azotobacter* isolates and their potential to promote sustainable crop production and agroecosystem resilience to climate change perturbations.

## Data Availability

The original data can be found as supplementary materials on Zenodo (https://doi.org/10.5281/zenodo.5760812) and on the Dryad Digital Repository (https://doi.org/10.5061/dryad.1vhhmgqv3). Further inquiries can be forwarded to the corresponding author. The accession numbers can be retrieved from https://www.ncbi.nlm.nih.gov/nuccore/?term=mw586872:mw586885[accn], https://www.ncbi.nlm.nih.gov/nuccore/?term=MW897940:MW897943[accn] and https://www.ncbi.nlm.nih.gov/nuccore/MZ066656 for all recovered sequences. The data are provided in the electronic supplementary material [35].
